# A Review of Emerging Technologies for IoT-Based Smart Cities

**DOI:** 10.3390/s22239271

**Published:** 2022-11-28

**Authors:** Md Whaiduzzaman, Alistair Barros, Moumita Chanda, Supti Barman, Tania Sultana, Md. Sazzadur Rahman, Shanto Roy, Colin Fidge

**Affiliations:** 1 School of Information Systems, Queensland University of Technology, Brisbane, QLD 4000, Australia; 2 Institute of Information Technology, Jahangirnagar University, Savar, Dhaka 1342, Bangladesh; 3 Department of Computer Science, University of Houston, Houston, TX 77204, USA; 4 School of Computer Science, Queensland University of Technology, Brisbane, QLD 4000, Australia

**Keywords:** Internet of Things (IoT), smart city, smart waste management, smart traffic light, smart parking, smart home, smart buildings

## Abstract

Smart cities can be complemented by fusing various components and incorporating recent emerging technologies. IoT communications are crucial to smart city operations, which are designed to support the concept of a “Smart City” by utilising the most cutting-edge communication technologies to enhance city administration and resident services. Smart cities have been outfitted with numerous IoT-based gadgets; the Internet of Things is a modular method to integrate various sensors with all ICT technologies. This paper provides an overview of smart cities’ concepts, characteristics, and applications. We thoroughly investigate smart city applications, challenges, and possibilities with solutions in recent technological trends and perspectives, such as machine learning and blockchain. We discuss cloud and fog IoT ecosystems in the in capacity of IoT devices, architectures, and machine learning approaches. In addition we integrate security and privacy aspects, including blockchain applications, towards more trustworthy and resilient smart cities. We also highlight the concepts, characteristics, and applications of smart cities and provide a conceptual model of the smart city mega-events framework. Finally, we outline the impact of recent emerging technologies’ implications on challenges, applications, and solutions for futuristic smart cities.

## 1. Introduction

**Figure 1:**
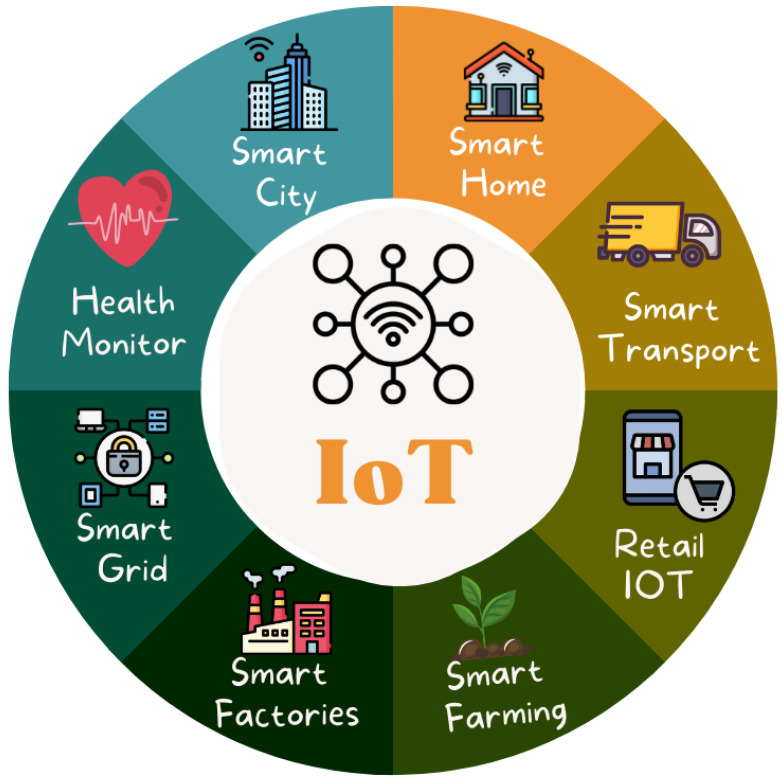
Applications of IoT for supporting smart cities.

The term “Internet of Things” (IoT) was coined in 1999, following the development of Internet-based technology in the 1990s [1]. The Internet of Things is a network of physical devices that may connect and interact in various situations, including social, environmental, medical, and user contexts. The IoT is an infrastructure made of real items, such as vehicles, buildings, and even simple electrical appliances, that are connected via the Internet to gather and exchange data with one another [2]. Due to the significant growth in population density in metropolitan zones, major infrastructure and services are necessary to suit the demands of city people. Over the next 30 years, it is anticipated that the world’s population will grow by more than 10%, with 70% of this by 2050, and the majority of people will reside in metropolitan regions [3]. As a result, countries are examining how to prepare their cities for the influx of people and the strain it will place on existing city infrastructure. Figure 1 depicts the applications of IoT needed to support such infrastructure.

The devices’ connection and communication abilities with the Internet and with one another, and the use of digital devices such as sensors, actuators, and mobile phones has expanded dramatically. Without human interaction, these “Things” can prioritise work, organise themselves, and interact with other “Things” [4]. The Internet of Things seeks to enhance the pervasiveness and immersion of the World Wide Web. The Internet of Things will speed up the creation of numerous applications that offer new services for businesses, consumers, and government agencies by utilising the immense quantity and variety of data created by “things”.

Smart cities employ these intelligent technologies for various purposes, such as energy consumption, pollution reduction, traffic management, lighting, and traffic control. This primary objective of smart cities can change our current perceptions of the world. We can assert that the Internet of Things will impact all facets of life, from simple daily duties to complex human emotions. Often, smart city applications and the surrounding environment are most advantageous to citizens [5]. Consequently, dynamic evolution and complexity characteristics necessitate a new methodological and modelling approach for mega-city planners. It must embrace technological improvements, transitioning from technology-driven (SC 1.0) through city government-driven, citizen-driven, Industry 4.0 (4G, 5G, electric cars, etc.) to artificial intelligence and cognitive computing (SC 5.0) [6,7].

**Figure 2:**
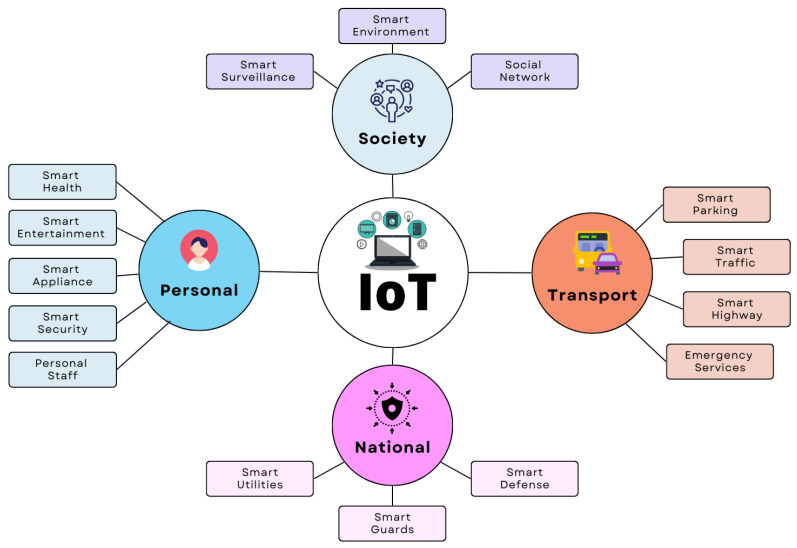
Interconnection of IoT components in smart city scenarios.

For good reasons, the concept of a “smart city” has gained momentum. First, urban areas are developing faster than ever since most new jobs are being created in these places. Second, many rural families are shifting to metropolitan regions to enhance their children’s educational opportunities [8]. This tendency has led to several significant problems. The infrastructure and facilities of urban regions must be expanded to support this population growth. Additionally, solutions should be devised for several issues, including those connected to the environment and transportation in urban areas. The smart city concept has been developed to address these problems. A smart city’s essential infrastructure requires and uses many sensors, support technologies, and background conditions in urban areas. The Internet of Things is regarded as one of the essential components for the successful implementation of a smart city [9]. IoT applications aim to ensure the critical links of Smart Cities, namely the regular operation of essential city systems, such as the water and electricity supply, fire alarms, and the proper operation of specialised processing, among other production capabilities [10]. Figure 2 shows the interconnection of IoT components relevant to smart cities.

This study offers an overview of smart cities’ concepts, features, and uses. We study the applications, challenges, and opportunities of smart cities in the context of utilising the latest technological developments, such as machine learning and blockchain (BC). This paper covers cloud and fog IoT ecosystems concerning IoT devices and architectures. In addition, we also incorporate security and privacy features, such as BC applications, to make smart cities more trustworthy and resilient. Moreover, we outline mega-event notions in smart cities and offer a conceptual framework. Finally, we incorporate the implications of current emergent technologies on future smart city issues, applications, and solutions. In this review, we make several contributions and lines of detailed discussions. The following is a summary of this paper’s main contributions:

1. We discuss the overall smart city concept in the context of cloud, edge, and IoT ecosystems.
2. We present IoT-enabled essential technologies, and architectures for overall discussions in different scenarios.
3. We present IoT-enabled machine learning, challenges, applications, and security and privacy concerns with recent emerging technologies including blockchain.
4. We provide an IoT-based framework for smart cities in the context of emerging technologies.
5. We discuss and outline the technical challenges and applications of the smart city in the context of recently emerging technologies.

The remaining sections of the review paper are structured as follows. Section 2 explores the history and rationale of smart cities. In Section 3, we explain IoT technologies for smart cities. Section 4 depicts the necessary IoT-based infrastructure of a smart city, including cloud, fog, and IoT ecosystems. Moreover, Section 5 illustrates the IoT designs from several viewpoints. Section 6 outlines IoT machine learning applications, scopes, and challenges, while Section 7 discusses security considerations, including blockchain applications. Smart cities experiences and a mega-event smart city framework is presented in Section 8. Section 9 discusses smart city applications. Finally, Section 10 highlights the smart city challenges and solutions followed by concluding remarks in Section 11.

## 2. Background

**Figure 3:**
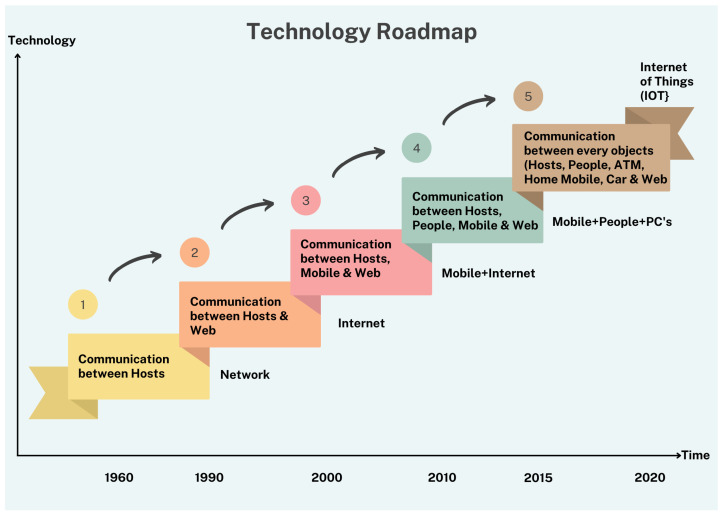
Technology roadmap of IoT.

Dynamic evolution and complexity characteristics necessitate a new methodological and modelling approach for mega-city designers. We present a brief history of mega-cities, the issues they currently face, and the emergence of smart cities. Following the introduction to the smart city comes a discussion of its characteristics and numerous generations. It has embraced technological improvements, transitioning from technology-driven (SC 1.0) through city government-driven, citizen-driven, Industry 4.0 (4G, 5G, electric vehicles, etc.) to artificial intelligence and cognitive computing (SC 5.0) [6]. Figure 3 and Table 1 depict the IoT technology roadmap and an overview of smart city models.

### 2.1. Smart City 1.0

Smart City 1.0 is the initial version of the Smart City initiative. SC 1.0 is defined as accepting technology businesses’ solutions in cities. These cities are usually criticised for their reliance on technology and the substantial impact of large corporations [6].

### 2.2. Smart City 2.0

Smart City 2.0 is acceptable when technology solutions to problems, such as pollution, sanitation, health, and transportation are developed in partnership with local citizens. Unfortunately, only a tiny percentage of residents are interested in participating in informal organisations and assemblies that make decisions.

### 2.3. Smart City 3.0

Smart City 3.0 is the third iteration of the smart city initiative. The government should support and clarify government-specific user requests while allowing public input.

### 2.4. Smart City 4.0

SC 4.0 embodies the greatest qualities of the past, such as the technical disruption of generation 1.0, the individualisation of generation 2.0, and the interactivity of generation 3.0.

### 2.5. Smart City 5.0

Smart City 5.0 can harmoniously balance all aspects of life and the opposing interests of many city stakeholders since it blends human cooperation and artificial intelligence. Smart City 5.0 provides a technique to facilitate “consensus” across several authorities and, more importantly, the general public [11].

Being on top of millions of sensing and communicating devices in real-time necessitates the recent rapid expansion of information and assets. These systems rely on Internet of Things technology and are devoted to utilising these resources in a fundamentally distinct and robust manner that allows them to reach their full potential. An assessment of the descriptions of a smart city by H. Arasteh et al. [2] serves as the introduction to a survey of the relevant literature that outlines the major aspects and characteristics of a smart city, as well as the IoT technology. The IoT and smart city concepts, as introduced by H. Rajab and T. Cinkelr [5], as well as their drivers and applications, are discussed in detail in the context of data integrity, management, and heterogeneity. The elements that influence implementing an information system that enables IoT and AI are discussed by Chatterjee et al. [7]. The benefits of employing IoT technology products should be known to potential customers, who should be informed that using this technology will make generating massive amounts of data more accessible.

Eunil Park et al. [8] mentioned the significance of IoT technologies on the technical roadmap of a smart city (TRM) to determine the importance and requirement of particular IoT technology components for a smart city. Amit Kumar Sikder et al. examined numerous IoT-enabled communication protocols and outline the smart lighting system that can be implemented in a smart city—illustrating how IoT-enabled smart lighting solutions may reduce indoor and outdoor electricity consumption by up to 33.33% [12]. Katherine Daz Barriga et al. [10] evaluated and analysed the available data, notably of generic and implemented structures in various smart cities around the world, and proposed a standard IoT architecture for smart cities. Lei Zhao et al. examined the edge resource allocation to reduce the average service response time, and their numerical results demonstrated the efficacy of the proposed algorithms [13]. Taher M. Ghazal et al. showed the use of IoT and WSNs-powered AI in the healthcare industry [14].

**Table 1:** Overview of smart city models.

ID	Sectors	Key Services	Advantages	Core Issues
[15]	Agriculture, Building	Built a testbed to simulate large-scale IoT deployments and produced CoAP and MQTT data	Cost-efficient	Did not support more request per seconds
[16]	Traffic Light system	Shows waiting time and vehicle density	Improve travel time, road safety, reduce traffic	—
[17]	Smart Parking	Availability of parking space	Save time, energy consumption	Does not take the weather or social events into account
[18]	Smart Home	Automation	High speed, multitasking	Low cost capacity
[19]	Smart building	Electronics embedded system	Improve quality of life	Affects monitoring tasks
[20]	Smart waste management	Management	Separate organic and recyclable waste	Less public spaces
[21]	Blockchain and smart city	Evaluate blockchain technologies	Provide reliability and secure services	High energy consumption
[22]	Smart Lighting	Evaluate various protocols	Save energy	Less security system, high installation cost
[23]	Cloud and smart city	Collecting and transmitting data	Energy management, waste management, reduce gas emission	—

## 3. IoT Technologies for Smart Cities

The Internet is the confluence point for the Internet of Things that employs industry-standard communication protocols. The Internet of Things is predicated on the pervasive presence of objects that can be measured, deduced, and understood and can modify their surroundings via multiple devices and communication technologies. Figure 3 shows the technology roadmap of IoT. Table 2 shows the comparison of IoT-based technologies for smart cities.

### 3.1. Message Queuing Telemetry Transport (MQTT)

The MQTT protocol is lightweight and suitable for millions of devices. Connecting devices over unreliable networks: MQTT for the Internet of Things uses QoS levels to assure message delivery to recipients even when connections between devices are unreliable. Compared to other protocols, this one is more adaptable.

### 3.2. Raspberry Pi

The Raspberry Pi is an inexpensive, credit-card-sized computer that connects to a computer monitor or television and is operated with a regular keyboard and mouse. The Raspberry Pi is an excellent choice for a central controller since it has an operating system, is capable of multitasking, has an integrated Wi-Fi module, and its hardware architecture supports four input peripherals.

### 3.3. Actuator

An actuator is a machine that changes energy and signals into motion in a system. Actuators are used in various devices, including ailerons on aeroplanes and electric door locks in cars.

### 3.4. Radio Frequency Identification (RFID)

The RFID reader is a portable or edge device that is network-connected. It transmits radio signals that turn on the tag. After being activated, the tag returns an electromagnetic wave to the antenna, which is then transformed into information. The RFID tag has an embedded transponder.

### 3.5. Sensor

A sensor is a device that detects environmental changes and responds with output to another system. A sensor converts a physical event into a readable analogue voltage (or occasionally a digital signal) that may be transferred for analysis or read by a human.

### 3.6. Global Positioning System (GPS)

The Global Positioning System, or GPS, is a satellite network that synchronises time, speed, and location for global navigation. GPS is a popular system found in cars, smartphones, and watches, and GPS facilitates navigation from point A to point B.

## 4. IoT-Based Computational Infrastructure

**Figure 4:**
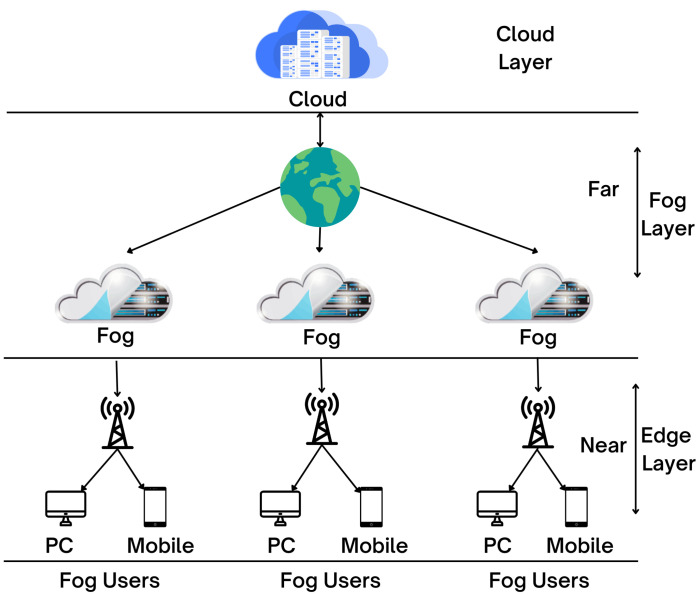
Cloud–fog–edge architectural layer as used in smart cities.

Smart cities rely on the latest innovation in cloud, fog, and edge computing to conduct data analytics as close as feasible to the point of data creation; fog and edge computing were created as extensions to cloud networks. The primary distinction between these two types is that edge computing is carried out directly on smart devices, whereas fog computing is carried out on the servers of the local network. Figure 4 shows the cloud–fog–edge architectural layer.

Green IoT is primarily concerned with the energy efficiency of IoT contexts. It refers to energy-efficient strategies for minimising or eliminating the glasshouse effect generated by present IoT applications. Fog computing and cloud computing are essential elements of the Green IoT implementation [29]. Computing in the fog and the cloud are crucial components of the Green IoT implementation. [30] illustrated how cloud and fog computing contribute to offering a variety of IoT services to end users.

### 4.1. Cloud Computing

The term “cloud computing” refers to a category of technology that enables data access from anywhere and at any time [31]. It is the trend of shifting software, platforms, infrastructure, or even hardware off-premises and utilising cloud service providers to deliver it to end users. Transmission Control Protocol/Internet Protocol (TCP/IP) networks are utilised in the cloud computing framework to connect servers with numerous scattered devices [32]. It manages storage deployment and accelerates data transport as a result privacy and data security are concerns if the data are not handled correctly to protect privacy.

### 4.2. Fog Computing

Fog computing is recommended as an alternative to cloud computing for all analytical workloads. Fog computing, which was developed to enable various IoT applications, can be viewed as a network-edge extension of cloud computing that can process data and analyse nodes near endpoints. Integrating cloud services with the network’s edge enables fog computing to execute computation, communication, and storage operations close to edge devices, which include IoT devices. This computing method improves low latency, limited network capacity, and privacy and security concerns. Al-Turjman and Malekloo investigated numerous fog computing applications and potential enabling technologies for sustainable smart cities in IoT environments, including the use of unmanned aerial vehicles (UAVs) and artificial intelligence (AI) techniques for caching data for fog-based IoT systems [33,34].

### 4.3. Edge Computing

Edge computing has been presented as a new paradigm meant to reduce and mitigate the downsides of cloud computing [35]. The phrase “edge computing” refers to a technique that extends and complements cloud computing by placing data processing as close to the manufactured devices as possible [36]. Analytical processing is performed locally at the edge rather than sent to the cloud to improve the processing of this gathered data. As a result, this type of computing increases bandwidth efficiency, reduces response time and network congestion, and consumes less energy. Edge computing systems must account for the possibility of an edge device being compromised and the data stored on the device being released. Different types of encryption can enhance privacy for edge computing applications [37].

## 5. IoT Architecture and Data Processing

**Figure 5:**
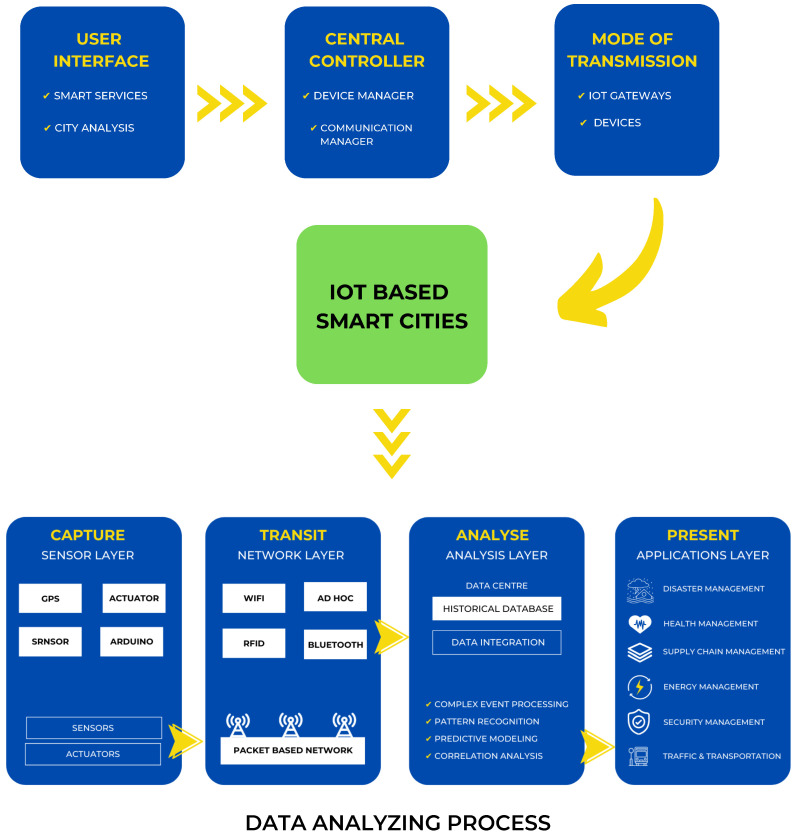
Architecture of IoT and data processing in smart cities.

Automation is a fundamental element of smart cities, and the primary objective of automation is to reduce human effort. In today’s world, remote-controlled systems are highly vital [18]. Consequently, the use of wireless technology in automation systems provides a variety of advantages that are not available with conventional networks. A city is considered “smart” if it integrates modern technology to facilitate daily tasks [38]. Figure 5 depicts the IoT architecture and data processing concepts relevant to smart cities.

### 5.1. IoT Architecture

In recent years, there has been a lot of research completed on IoT architecture with regard to how smart functionalities are carried out. The architecture of IoT consists of 3 layers:

#### 5.1.1. User Interface

The user interface is used to direct or regulate the city’s electrical appliances. For instance, by tapping a button on a smartphone, utilising voice instructions to control, etc.

#### 5.1.2. Mode of Transmission

A connection can be wired or wireless to send control signals. Ethernet, MQTT, Wi-Fi, Bluetooth, and other protocols are utilised for data transfer and control. The architecture greatly benefits from the middleware layers. Middleware packages IoT infrastructure for consumers by combining common abstraction methods and functions [39].

#### 5.1.3. Central Controller

The central controller is a piece of hardware that links the user interface and the transmission mode. Sometimes this central controller is also connected to every piece of electrical equipment, such as a Raspberry Pi, Sensors, Actuator, or MQTT.

### 5.2. Data Processing

To allow IoT functionalities, numerous data mining and processing approaches have been proposed. The four stages of the data operation process are commonly divided into sensor layer, network layer, analysis, and implementation/application layer [40].

#### 5.2.1. Sensor Layer

IoT data gathering can be divided into identification and sensing. The address and ID of objects in a network are identified as part of the identification process. In a network, an object’s ID might not be unique. In this scenario, the object is precisely identified using an address. Utilising sensing tools, such as actuators, wearable sensors, smart sensors, cellphones, and other sensing devices, data are collected during the sensing process [41].

#### 5.2.2. Network Layer

The communication protocols used in IoT-based networks have a considerable impact on how IoT data are delivered. Currently, a wide variety of communication protocols are in use, including IEEE 802.15.4, Wi-Fi, and Bluetooth. In ad hoc settings, Wi-Fi is another important data transport technique used to transfer data between smart devices [41].

#### 5.2.3. Analysis Layer

To facilitate IoT data analysis, a number of software platforms and operating systems (OSs) have been developed. The IoT is powered by a variety of open source and commercial OSs, unlike PCs and mobile devices. It is common practice to manage hardware resources, host applications, and process data in real-time using real-time operating systems (RTOSs) [41].

#### 5.2.4. Application Layer

The development of trustworthy IoT solutions in various sectors, including transportation, automotive, healthcare, and the computational component of IoT, is complemented by cloud platforms offering beneficial, application-specific services [38]. IoT technologies are anticipated to be used in large-scale networks with thousands of devices and coverage areas that extend for several kilometres. Smart city architects and providers understand that to utilise IoT fully, cities must give scalable and secure IoT solutions incorporating effective IoT systems [42].

## 6. IoT and Machine Learning

**Figure 6:**
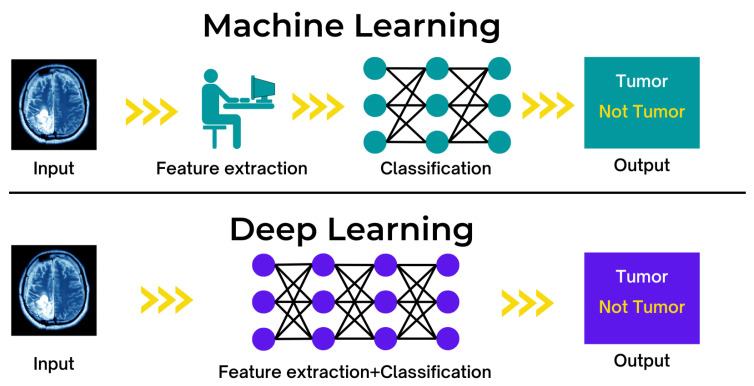
Function of machine learning and deep learning in a healthcare context.

Applications have evolved due to the Internet of Things (IoT), and connected devices have enabled their use in numerous aspects of a modern city [43]. As the amount of acquired data increases, machine learning techniques are employed to enhance an application’s intelligence and capabilities [44]. To address various security concerns, machine learning (ML) techniques that embed intelligence in IoT devices and networks are employed [45]. The Internet of Things offers various applications across all industries, empowering and increasing human life, such as AI-powered IoT for consumers to listen to music on smartphones while driving or performing other tasks. Headphones with Internet of Things sensors can track heart rates and use artificial intelligence to predict emotions; the smartphone may select the world’s best song from those stored. ML can aid intelligent machines and devices in making positive inferences about the world. Smart devices can modify or automate a state or behaviour based on knowledge, considered a critical component of an IoT solution. Figure 6 shows the function of machine learning and deep learning.

### 6.1. Applications

ML techniques have been implemented inmany applications, including classification, regression, and density estimation. Various applications, such as computer vision, fraud detection, bioinformatics, virus detection, authentication, and speech recognition, employ machine learning methods and techniques [44]. Artificial intelligence is the development of machines clever enough to carry out tasks independently. IoT devices generate vast quantities of data to develop cutting-edge AI models. The combination of IoT and AI can significantly enhance the quality of human life. Machine learning is intelligent procedures using examples or previous experiences to optimise performance criteria. Specifically, ML algorithms develop behaviour models by applying mathematical techniques to large datasets. These models serve as the basis for future estimates based on the newly added data [45].

#### 6.1.1. Health Disease Prediction

Heart disease is one of the leading causes of death in the modern world. Mohan et al. [46] suggest a novel approach to increase the precision of cardiovascular disease prediction by identifying key variables using machine learning techniques and providing an improved performance level with an accuracy level of 88.7% when using a mixed random forest and linear model to predict heart disease. Various DL models are performed on these data to forecast potential disorders. Combining deep reinforcement learning with medical Big Data, generated and acquired through the medical Internet of Things, holds promise for computer-assisted diagnosis and therapy [47]. Using an Internet of Things (IoT) architecture, Convolution Neural Networks (CNN) are utilised to categorise strokes from CT scans to distinguish between a healthy brain and an ischaemic or haemorrhagic stroke [48]. Given the incidence of lung malignancies, attention has been directed to the possibility of deep reinforcement learning for early lung cancer diagnosis [49].

#### 6.1.2. Road Accident Prediction

According to the United Nations (UN) 2030 agenda, the implementation of the transport system would be made possible by digital technologies, such as the Internet of Things (IoT) and artificial intelligence (AI). Implementing these digital technologies on highways provides a dependable, superior, more intelligent, and renewable energy-powered experience to highway users. Several categorisation models, including Logistic Regression, Artificial Neural Networks, Decision Trees, K-Nearest Neighbors, and Random Forest, have been used to predict the severity of an accident.

#### 6.1.3. Industry Maintenance

In the business world, maintenance is a well-known approach consisting of all the operational methods and steps required to ensure machine availability and prevent a machine-down failure. Calabrese et al. [50], noted that designing and developing an embedded intelligent system to track and predict the health status of the machine is one of its critical problems. The latest industrial revolution, termed “Industry 4.0,” promises to increase the productivity of industrial systems by improving resilience, flexibility, and adaptability. Earlier efforts to improve the performance of typical industrial systems focused on scheduling techniques. Brik et al. [51] proposed a tracking tool for Industry 4.0 system disruptions focusing on localising resources. Consequently, a machine learning technique is used to create a prediction model of resource localisation by incorporating actual task scheduling.

#### 6.1.4. Smart Agriculture

To increase the quantity and quality of crop production from crop fields, the agriculture industry will implement machine learning and IoT data analytics. The power and capability of agricultural computing technologies, such as wireless sensor networks, the Internet of Things, data analytics, and machine learning. Akter and Sofi [52] proposed integrating data analytics and machine learning into an IoT system to predict apple disease in apple orchards in the Kashmir Valley. Maduranga and Abeysekera [53] stated that IoT-based smart agriculture has attracted the interest of researchers who have used IoT and machine learning (ML) technologies to conduct groundbreaking research. The IoT’s primary objective in agriculture is to automate all agricultural operations and methods to maximise productivity.

### 6.2. Machine Learning on Security and Privacy for Smart Cities

The residents of these smart cities are connected via smartphones and high-tech equipment with the Internet of Things integration, enhancing lifestyles which also raise concerns about information security, privacy, and unauthorised data access. Modern smart cities result from significant advances in information and communication technology. Smartphones and other high-tech devices with Internet of Things integration connect the residents of these smart cities, boosting their quality of life and providing unfathomable benefits. Deep learning and associated technologies effectively remedy these security issues in applications that employ Big Data and IoT technology [54]. In applications employing Big Data and Internet of Things technologies, deep learning, and related technologies have proven effective in mitigating such security risks [55]. Malware insertion is the most common threat [56]. Using the operational device codes (OpCode) sequences, a deep Eigenspace learning method for identifying IoBT malware is proposed. IoT botnet attacks have proliferated because IoT devices are more susceptible to compromise than desktop PCs. To mitigate this risk, advise employing DAE to identify aberrant network activity from compromised IoT devices [57].

### 6.3. Challenges of Using Machine Learning with IoT

Several new challenges must be considered while creating a smart city ecosystem. Due to the Internet of Things devices’ involvement in many applications, user security, and privacy have become significant problems. The generation of reliable outputs from extensive, complex databases using machine learning algorithms allows for predicting and detecting vulnerabilities in IoT-based systems [58].

#### 6.3.1. False Data Detection

Threats to deep learning (DL) models come in a variety of forms. These dangers impact these models’ functionality and reduce their precision, reliability, and validity. False data injection is a threat that uses sensors and IoT devices worldwide to broadcast erroneous measurements and data. False data injection seeks to deceive analytics process operations, producing inaccurate findings, recommendations, and forecasts [59].

#### 6.3.2. High Cost

A significant issue still lies in choosing the right technology to successfully and efficiently integrate with smart city services. Another significant problem is getting administrative organizations to adopt and use these technologies in their regular services and departments. Preconceived ideas about the initial cost of such technology would raise the budget; however, once implemented, it would produce superior economic results [55].

#### 6.3.3. Reliability

Reliability is a fundamental problem with neural networks that has not received much attention. Because neural networks are not completely precise, employing a trustworthy platform for delicate applications, such as self-driving cars and cancer diagnosis, is still necessary to guarantee the most accuracy and dependability [60].

#### 6.3.4. Data Labeling

Finding an appropriate model to handle data flowing from various IoT devices is complex, and adequate input data labelling is also challenging. For example, in the case of healthcare applications, the training model must be highly accurate to extract the useful features from the data and learn efficiently, as false positives in such models could have disastrous repercussions [44].

#### 6.3.5. Lack of Multitasking Capabilities

DL models are trained in a particular application domain and are highly specialized. These models are only valid for solving a single sort of problem for which they were developed. Re-evaluation and retraining of the model are critical whenever there is a change in the definition, status, or nature of the problem [44].

## 7. Security, Privacy, and Blockchain

In this section, we first discuss the issues and challenges related to privacy, security, and blockchain integration in the context of smart cities. Then, we discuss a few solution approaches that address some of these issues.

### 7.1. Security and Privacy Issues

Security is one of the significant challenges, given how quickly IoT-connected devices are growing. The smart grid is vulnerable to significant attacks because the gadgets are online. Because the devices are online, the smart grid is susceptible to severe attacks [61].

#### 7.1.1. Lack of Encryption

Even though encryption is a great way to prevent thieves from acquiring data, it presents one of the significant security concerns for IoT. These drives are used for a typical computer’s processing and storage power, and the ultimate result is increased attacks where hackers may swiftly alter the security algorithms [62].

#### 7.1.2. Data Security

The data backup provided by the service provider is a substantial security issue. It is the responsibility of SaaS providers to manage data security while the plain text is processed and stored in the cloud, preventing unauthorised users from accessing sensitive information [63].

#### 7.1.3. An Attack on Virtual Machines

**Figure 7:**
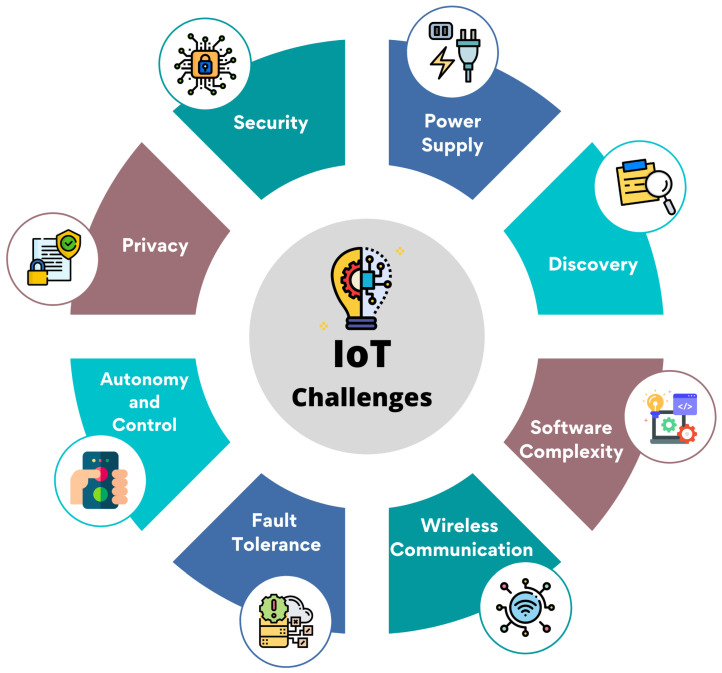
Challenges of IoT relevant to smart cities.

Virtual Machine (VM) security is of the utmost significance, and any security breach could result in the collapse of the entire IoT environment [63]. Figure 7 depicts the IoT-related challenges of smart cities.

#### 7.1.4. Interoperability

A fragmented proprietary IoT technology deployment environment may limit users’ value. Users might not like buying goods and services where there is little freedom, and there are concerns about dealer lock-in, even though perfect interchange across products and services is not always attainable [64].

### 7.2. Blockchain Challenges and Issues

There are several challenges in integrating BC with smart city aspects. We discuss and elaborate these issues and challenges in the following sections.

#### 7.2.1. Uncertainty

Blockchain technology is still a relatively new concept, and only a small number of installations have been successful with existing technology. As a result of the scarcity of workable BC models, this is a hurdle because it increases uncertainty.

#### 7.2.2. Privacy of Citizens

Blockchain has a privacy issue in that users can only employ pseudonyms to be completely anonymous, and making oneself untraceable and invisible is the aim of anonymity. Due to the transparency of the BC, all transactions are visible to the public, and details such as the sender, receiver, and amount values are evident to all network participants. This is accurate, although each BC user is associated with the public pseudonymous address [65].

#### 7.2.3. Standardization

There is a lack of uniformity in the design of BC network which creates challenges during implementation because the platforms used for implementation involve several partners from different economic sectors. A robust integration layer and BC standardisation are still needed for various use cases [66].

#### 7.2.4. Legal Uncertainty

Blockchain-based applications sometimes lack a thorough understanding of the laws and regulations governing insolvency, failure scenarios, and fraud. Several complicated cross-border jurisdictional challenges can be presented by BC nodes [67].

#### 7.2.5. DDoS Attack

This attack intends to overwhelm connections so that smart city network users cannot connect. A target is inundated with anonymous requests, which inhibits the completion of legitimate requests. The connectivity of city websites and servers can be seriously impaired by these botnet attacks, which can seize control of IoT devices and consume enormous amounts of bandwidth at Internet gateways. These threats occur when the economies of smart cities are thriving, and they are taking measures to attract investments worth more than USD 150 billion globally by 2022. The cunning cybercriminal uses malware code to cause problems to evade detection and limit users’ broadband usage [68].

### 7.3. Solution Approaches

In This section, we discuss several security solutions in the context of smart cities.

#### 7.3.1. Encryption-Based Solutions

Encryption aims to completely conceal data but is only as effective as the employed algorithm and cryptographic key. Encryption may be used in each tier of a smart city’s architecture, making it a versatile technology. Encryption sets the groundwork for its classification, as numerous other approaches rely on it to achieve privacy objectives. ABE-Cities, is an urban sensing encryption system that uses attribute-based encryption to address the aforementioned challenges and ensure fine-grained data access control [69]. In another approach, SmartEdge, an end-to-end encryption framework for smart city applications, was developed using a lightweight symmetric encryption technique by SVM training approach, where IoT data are encrypted before being recorded on a distributed ledger [70].

#### 7.3.2. Blockchain-Based Solutions

**Figure 8:**
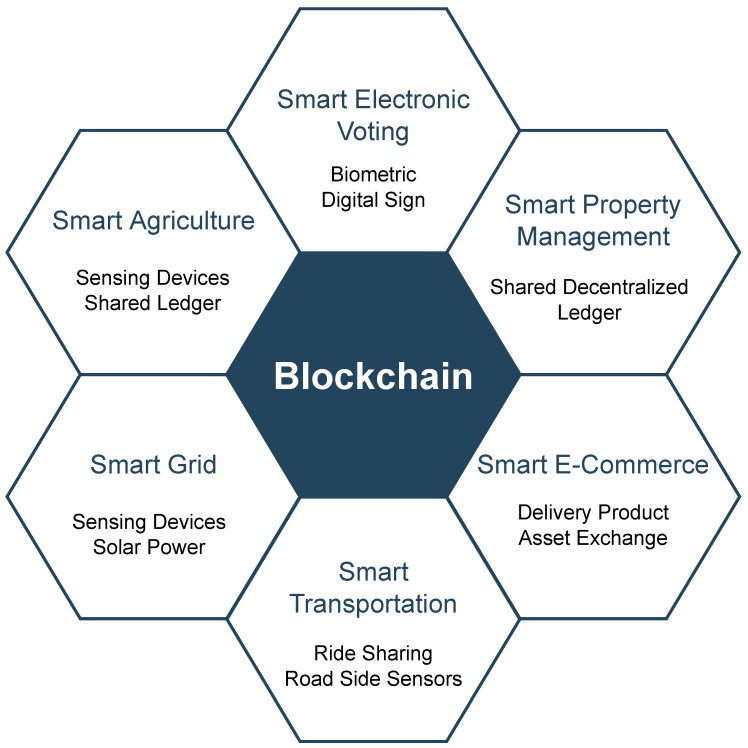
Blockchain-based applications in smart city aspects.

In this section, we review recent BC-based research in smart city ecosystems. This part aims to evaluate and categorize the most recent scientific discoveries as potential remedies for BC-based smart cities [71]. Figure 8 shows the smart city aspects of BC in terms of use cases and application domains.

##### Smart Electronic Commerce

In electronic commerce, sellers and buyers trade assets on platforms. In current e-commerce systems, trusted third parties (TTP) handle the delivery of traded items. BC makes it possible for parties to communicate directly and without any basis for trust during transactions. BC technology and smart contracts can be used to centralise online retailers from such an e-commerce ecosystem. A dual-deposit escrow system was suggested as a solution to the issue of the buyer and seller for the sale of a digital good. There is an issue with payment and delivery of genuine digital goods [72]. Hasan and Saleh [73] propose a Proof of Delivery (PoD) framework for physical assets on the BC employing a secure, open logistics management solution to deliver tangible goods through a single carrier or several intermediary transporters.

##### Smart Electronic Voting System

In a smart city, e-governance aims to leverage ICT to automate the governance process. At the federal, state, and municipal levels, electing representatives of the people by voting is a democratic process. The integrity of the government workers who run the polls is essential to the integrity of paper voting. Aside from this, ballot-based voting has a variety of other disadvantages, such as high costs, time requirements, anomalies, pre-poll rigging, inaccurate vote tallying, and low voter turnout. Electronic voting, a form of polling, uses digital technology for voter verification using biometrics software systems. An Electronic Voting System (EVS) stores the votes and can process the election result immediately [74].

##### Smart Transportation System

Intelligent transport systems (ITS) integrate cutting-edge technologies, such as computing devices, sensor networks, wireless communications, and electronics to make transportation systems effective, secure, convenient, affordable, and connected. The smart transportation system [21] includes traffic signal management systems, the Speed Detection Camera System (SDCS), automatic number plate recognition, CCTV systems for real-time surveillance, and traffic ticket administration systems. BC can be used in the ride-sharing transportation economy. Laurent et al. [75] propose a framework for BC-based ITS (B-ITS) with seven layers. Vehicles can be registered in the BC at the physical layer using IoT as computer and communication equipment. The data layer deals with the safe addition of data in the form of blocks.

##### Smart Grid

The “smart grid” is responsible for producing and dispersing electricity under automated supervision [76]. It is made up of electricity generators, renewable energy sources, smart metres, transmission lines, and other intelligent equipment. Renewable energy sources (RES), such as wind and solar power generation, have introduced so-called prosumers to the energy trading markets. The smart grid’s local power generation for on-site use has comparatively little transmission loss. Blockchain provides a framework for exchanging energy in the smart grid [77].

##### Smart Property Management

Real estate deals with public, private, and commercial properties on an ownership or rental basis. Smart real estate management (SREM) uses cutting-edge modern technology to effectively handle commercial real estate transactions in a user-centric, safe, privacy-preserving, and sustainable way. Blockchain technology can create new business models in the real estate industry by addressing the requirements of corresponding parties, such as customers, real estate agents, the government, and regulators, within a shared decentralized ledger. BC can readily address the problems with land registration to stop property fraud. The immutable BC provides the complete ownership history of a property and the verification and certification of related transactions [78].

## 8. Smart Cities Experience and Mega Events

In this section, we will focus on worldwide smart cities experience and the framework of a mega-city in the context of mega-events.

### 8.1. Experiencing Smart Cities Worldwide

Singapore, Zurich, and Oslo are the world’s brightest cities to the 2021 Smart City Index. The Institute for Management Development at Singapore University for Technology and Design (SUTD) conducts an annual evaluation that ranks cities based on economic and technological facts and citizen perceptions of how “smart” each city is. Each year, more and more towns are added, with Lausanne, Switzerland, joining the research for the first time in 2021 and entering the list of smart cities at number five. Leeds and Glasgow in the United Kingdom, Bordeaux and Lille in France, Kiel in Germany, Medina in Saudi Arabia, Istanbul in Turkey, and San Jose in Costa Rica are among the most recent additions to the list. Table 3 shows the strategies of cities around the world.

### 8.2. Smart City Framework Perspective of Mega Events

Organising a large-scale event is a complex endeavour that requires the cooperation of all relevant organisations, authorities, and stakeholders. Mega-events are noteworthy, attract a large audience, and occur regularly in multiple cities. Barcelona, Sydney, Beijing, and London all experienced these benefits of hosting the Olympic Games. Hefnawy et al. [79] present a suitable platform for information and knowledge sharing, both within the same event and with other events of a similar nature. Vertical service provisioning and horizontal integration across multiple sectors enhances secure and reliable platform integration to connected smart devices for a wide range of applications and services [80].

**Figure 9:**
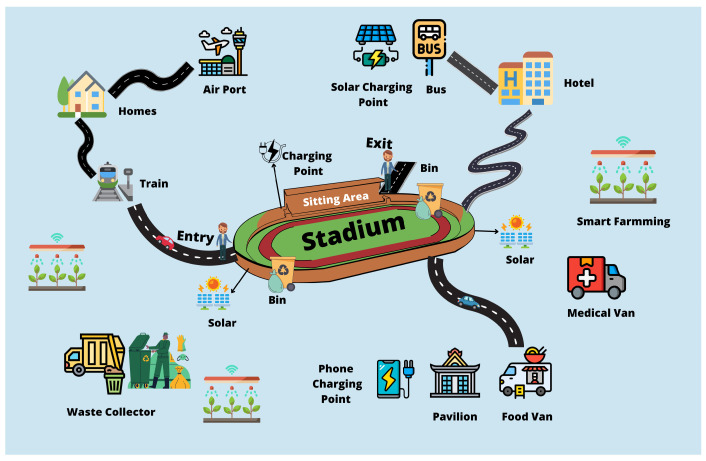
Conceptual overview of a mega-event in a smart city.

We propose a framework based on lifecycle management and smart city principles to enhance the management of large-scale events. Figure 9 shows the conceptual overview of a Megaevent. A new set of computer and network paradigms and technologies has emerged in recent years, including Software-defined networking (SDN), IoT, network function virtualisation (NFV), LTE/5G high-speed wireless technologies, cloud computing, big data, cognitive management, machine learning, and blockchain can provide a fresh perspective on the development of the smart city.

**Figure 10:**
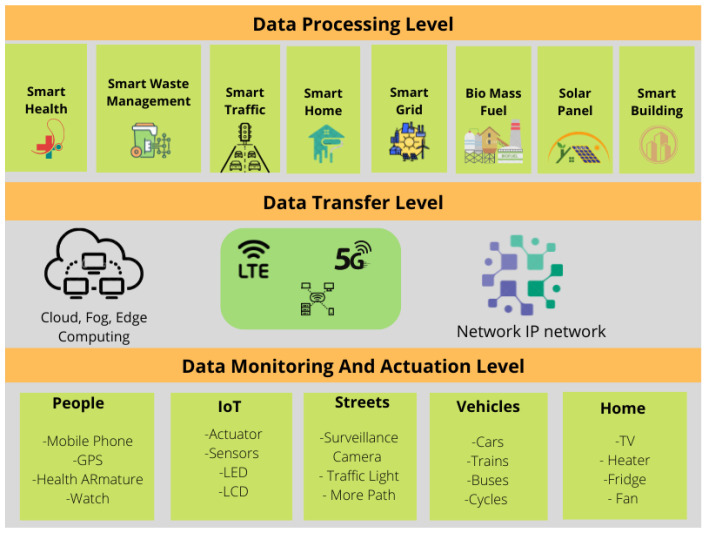
Smart city conceptual framework.

Figure 10 shows our the framework of a smart city. The physical components level, also known as the data monitoring and actuation level, contains all the devices, sensors, tools, apparatus, and parts that comprise the numerous “smart” systems, services, and applications. The communications level contains all possibilities for network-based technologies that facilitate communication between applications and physical components. All “smart” systems and urban planning improvements go within the application level (data processing level) [81,82].

## 9. Applications for Smart Cities

The following are some of the application focus areas for developing a smart economy. Any form of functional government must employ ICT tools to increase public participation. Giving social and human capital a variety of technologies and tools will surely make them smarter. To build a smart environment, less dangerous or damaging gas emissions and alternative forms of waste disposal should be implemented with minimal environmental impact and conserving natural resources [19]. Table 4 represents the major components of an Internet of Things-enabled smart city environment and an overview of IoT applications, respectively.

### 9.1. Waste Management System

The core of a smart city is a smart environment, which is mostly employed for systems dealing with environmental degradation. Urban trash management is a routine task requiring a significant amount of labour and impacts social, economic, environmental, and efficiency variables [83]. Waste management has a substantial impact on the quality of life of the populace. In the trash can, sensors, GPS, and LED could be used to manage trash. The sensor automatically notifies the operator when to collect the trash can and the route to get there while ignoring traffic. When the trash can is complete, an alert is sent to the operator, who then relays the location to the smartphone-equipped waste collector. The collector then travels to the spot, gathers trash, and replaces the bin [24].

### 9.2. Smart Parking System

**Figure 11:**
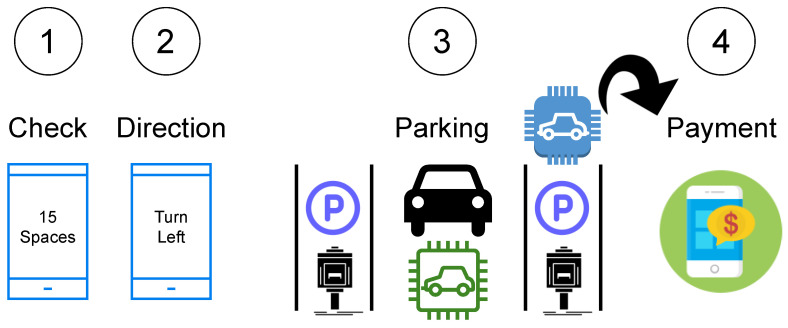
Elements of a smart parking process for a smart city.

Drivers are informed of the availability of parking spaces at various places by the car parking information system (CPIS). Parking space availability is a challenge that is time-dependent. Time series analysis techniques were used to assess the sequential nature of the information on parking slot occupancy and duration of occupancy [17]. Figure 11 shows the smart parkingsystem.

### 9.3. Smart Traffic Light System

**Figure 12:**
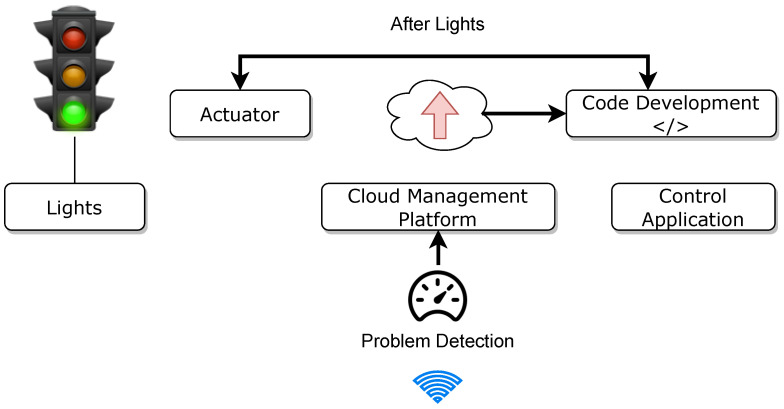
Smart traffic lighting system in a smart city.

Smart traffic lights lead to a citywide decrease in air pollution. Light fading does not happen with the advantages of brightness control, including energy savings and avoiding accidents caused by blinding lights. Connecting car owners and alerting them of impending road conditions is a highly beneficial IoT application. To detect oil spills and potholes, computer vision-enabled cameras scan the road often during the transition from day to night and when the weather begins to deteriorate. It may be possible for roadside light sensors to detect these signals and activate or deactivate the lights accordingly. When a motorist exceeds the regular speed limit while operating a vehicle, the cloud detection system identifies this as an issue, and the authorities take action against the driver. Figure 12 shows a smart traffic lighting system.

### 9.4. Smart Building System

**Figure 13:**
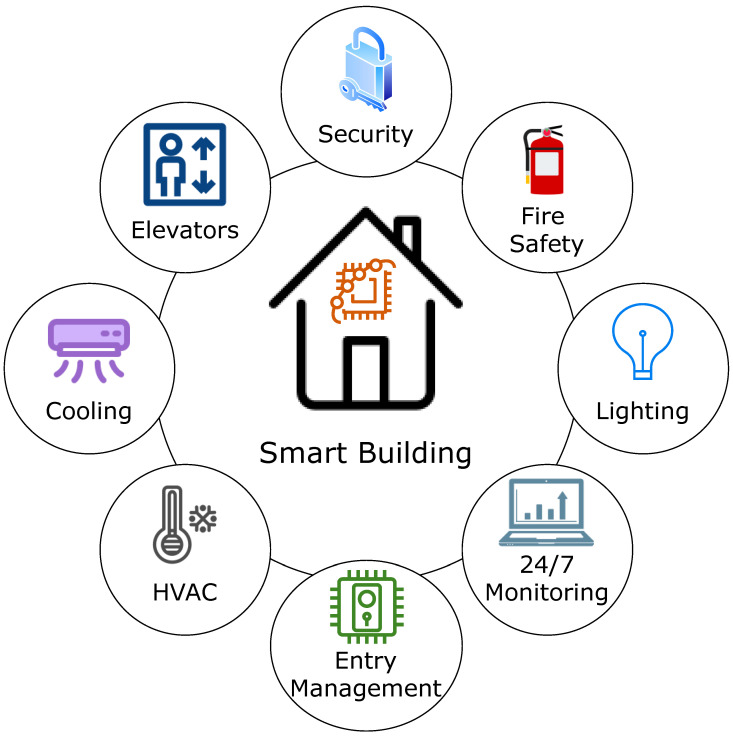
Elements of a smart building.

IoT home automation refers to the ability of internet-connected, electronically controlled technologies to operate household appliances [84]. Support for IoT-enabled smart buildings is a useful and cost-effective user-level IoT application. It is feasible to install complex heating and lighting systems, alarms, and home security controls linked to a single hub and operated remotely via a smartphone app. The primary objective of automation is to reduce human effort. We can utter commands such as “FAN ON” and “LIGHT ON” to activate electrical appliances [18]. Figure 13 depicts features of an intelligent building.

**Table 4:** Overview of IoT applications.

Field	Id	Algorithm	Goal	Advantage	Technology
Smart Waste Management System	[27]	WSN, IoT	Watch the trashcan	Lessen the challenges associated with cleaning procedures	Ultrasonic Sensor, Arduino UNO, Wi-Fi
	[83]	Machine Learning, graph theory	Show the amount of waste in cans	Inexpensive, replaceable	LoRa
	[85]	Machine Learning, IoT	Waste collection and decomposition	Reuse energy	KNN
Smart Building System	[13]	IoT, plug and play learning framework	HVAC controls	Avoiding a building-by-building arrangement	Sensor
	[84]	IoT	Energy management of buildings	Lower installation cost	Power over Ethernet (PoE)
	[86]	WSN, IoT	Cost-effective, versatile, and reliable wellness sensor networks	Mobility of objects inside the home to predict a person’s health	ZigBee, A301, LM35 IC
	[87]	Data fusion Techniques	Establish a framework with spatio-temporal data	Low-cost hardware and software, save energy	Arduino microcontroller, WiFi
Smart Parking System	[88]	IoT based Cloud	Availability of parking place is tracked	Bolster parking infrastructure	Ultrasonic Sensor, raspberry pi
	[89]	Cloud+IoT	Automatically locate a cost-effective parking spot	increases parking attempt and reduces user waiting time	WSM, RFID
	[29]	Cloud based hybrid-parking model	Reduce the gridlock caused by parking issues	Offer the public the highest-quality services while making a profit	Ultrasonic Sensor, RFID
	[90]	E-parking system	Check for parking space availability and reserve a spot	Seeing automobiles parked improperly in the parking space	Wi-Fi
Smart Traffic Light System	[16]	IoT and Adaptive Neuro Fuzzy Inference System (ANFIS)	A better traffic situation	boost the drivers’comfort and safety	Arduino UNO
	[91]	GSM	Guarantee safety and avoid energy wastage	automated streetlight ON/OFF switching	Microcontroller MSP430, LDR sensor.
	[92]	Arduino board	To reduce the gridlock caused by parking issues	better performance	LED, HID lamps.

## 10. IoT-Based Smart City Challenges and Solutions

We have discussed several specific topic-based aspects of challenges and solutions in previous relevant sections. However, in this Section, we will outline in general IoT-based smart city challenges and solutions.

### 10.1. Challenges

**Figure 14:**
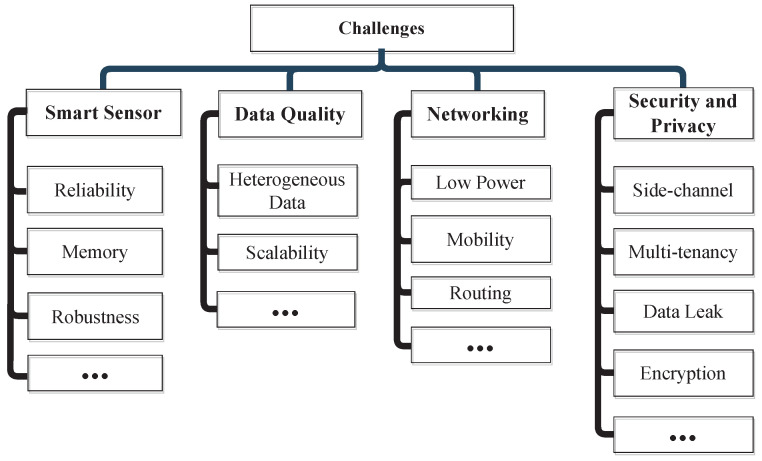
Smart city challenges.

This section addresses the typical issues and challenges brought on by the implementation of IoT-based smart cities [5,7]. Figure 14 shows the smart city challenges. Table 5 shows the challenges and solutions of the smart city.

#### 10.1.1. Assuring Data Quality

Users may be confident that the complete dataset has been merged since data integrity and quality emphasise describing the properties and values of the data (metadata, context). As a result, costs may be efficiently searched, shortened, and filtered. The discussion of IoT applications for smart cities requires both quality and integrity. It enables better bin identification, management, and monitoring in waste management systems. Moreover, if data integrity and quality are not guaranteed, the gathered data may be tampered with, and power grid operators may make the wrong decisions.

#### 10.1.2. Security and Privacy

All the data can be collected and analysed on a single IoT platform, exposing the system to various dangers (e.g., cross-site scripting and side-channel). Moreover, such a system is susceptible to severe faults. In addition, this system’s multi-tenancy may disclose security vulnerabilities and lead to data loss. For instance, electronic signatures used to create an e-democracy system are susceptible to side-channel attacks.

#### 10.1.3. Reliability

The IoT-based system has experienced a few dependability problems. For instance, vehicle communication is not dependable enough due to their mobility. Additionally, the proliferation of smart gadgets will provide certain dependability issues in terms of their failure [98].

#### 10.1.4. Smart Sensors

Smart sensors are the hardware components that collect data in smart cities. These devices are manufactured by many diverse organisations utilising distinct sensing procedures, measurement standards, data formats, and networking protocols. These devices must be able to exchange data, schedule tasks, and aggregate data for implementing smart cities. Robustness and dependability are additional obstacles for intelligent sensors. Reliability and robustness are phrases used to define the dependability and precision of the IoT system. Therefore, the IoT system must provide its users with a seamless experience. This requires accurate and timely responses to service requests from application users. Every resident of the smart city must receive superior services.

### 10.2. IoT-Based Solution

IoT technologies are a crucial component for progressing smart city plans to realise the concept of a smart city. To manage the diverse properties of IoT technology, the following solutions should be necessary.

Several strategies, such as closest neighbour search, colony optimisation, genetic algorithm, and particle swarm optimisation, have been developed to optimise waste management. Khoa et al. [83] describe a novel waste management system that adequately predicts the likelihood of trash can overflow. For vehicle routeing problems, two submodels are developed. The first submodel may estimate the threshold waste level (TWL) parameter using current IoT-based sensors for traceability and real-time data collection. To generate positive social and environmental outcomes for WMS over the long run, selecting a collection path that is both efficient and distinct is essential. To increase recovery value and reduce visual pollution in the second model, waste sorting and transportation to the recovery value centre are considered. The KNN machine learning technique is utilised for combinations of three sensor data to generate an alarm message [85].

Khanna and Anand [88] describe an IoT-based, cloud-integrated smart parking system that uses an on-site deployment of an IoT module to track and communicate the availability of each parking space. In addition, a smartphone application permits users to determine the availability of parking spaces and reserve a spot accordingly. The technology recognises automobiles in indoor and outdoor parking lots using ultrasonic and magnetic sensors. In parking lots, BLE-enabled wireless sensor nodes support a vehicle-locating service [99]. Pham et al. presented a method for calculating the user parking cost based on the parking lot’s distance and the total number of slots. Based on new performance indicators, the system assists clients in selecting a free parking spot at the lowest price.

In a smart city, the lighting system combines sophisticated sensors and communication channels to create a smart lighting system (SLS). An SLS is intended to provide an autonomous, more efficient lighting management system. A NodeMCU V3 with Wi-Fi capabilities, a passive infrared sensor to detect room occupancy, a relay switch to handle lights switching, and an IoT-based controller device with a cloud-based system are used to switch, manage, and monitor the lights in university classrooms. The developed applications serve as a controller, a management system to build appropriate class schedules, and a monitoring system to indicate the usage duration and overall energy spent by lights [100]. Khoa et al. [83] showed how using the Secure Hash Algorithm 256 (SHA-256), an authentication system verifies each interaction between a particular device and a WebServer by encrypting the login, password, and token can enhance security efficiency. Using this architecture, light switches, guest attendance monitoring, and automatic burglar alarm systems might be constructed and integrated into the infrastructure of any smart city.

Sushanth and Sujatha [101] included the creation of a system that can use sensors on an Arduino board to monitor temperature, humidity, moisture, and even the movement of animals that could destroy crops, and in the event of discrepancies, send SMS notification and a notification on the application created for the same to the farmer’s smartphone via Wi-Fi/3G/4G. The cellular-Internet interface of the system’s duplex communication link enables Android application-based data analysis and irrigation schedule programming. Gondchawar et al. [102] provided an intelligent, GPS-enabled remote-controlled robot is provided that may be utilised for weeding, spraying, detecting moisture, scaring away birds and other animals, and keeping watch. The third part of intelligent warehouse management is monitoring the facility’s humidity, temperature, and theft. Any remote smart device or computer with Internet access will be used to control these operations, which will be carried out by connecting sensors, Wi-Fi or ZigBee modules, cameras, and actuators to a microcontroller and a Raspberry Pi.

Sikder et al. [12] described a smart agriculture IoT system that incorporates reinforcement learning and consists of four layers: agricultural data collection, edge computing, agricultural data transmission, and agricultural data cloud storage. The mentioned technology merges agricultural production with cutting-edge computer technologies, most notably cloud computing and artificial intelligence, to increase food production. Deep reinforcement learning, the most powerful artificial intelligence model, is linked to the cloud layer to make quick, intelligent decisions, such as estimating how much water must be irrigated to optimise the environment for crop growth.

## 11. Conclusions

This study focused on IoT as a platform for multiple smart city applications, considering the recent emerging technological trends with IoT, including AI and machine learning, cloud edge IoT ecosystems with blockchain, and security aspects. We also considered waste management, smart parking, smart building systems, and traffic light systems that consume less energy. Because installing IoT infrastructures could present various opportunities, followed by a few practical applications to facilitate the development, enhancement, and improvement of daily chores. We introduced smart cities in machine learning-based applications, challenges, and solutions, including security and privacy-preserving aspects of smart cities. We also considered the sustainable smart city’s cloud–edge–IoT ecosystems focus on IoT architecture and technologies, including IoT devices and protocols. We also provided details on IoT applications, challenges that may occur when providing detailed instructions on IoT system design, and a solution to some of the most significant issues, such as management, coordination, and the implications of blockchain technologies. We also provided a framework forthe mega events perspective and outlined the applications and challenges of future smart cities. We summarized and synthesized the IoT-based smart city implementation utilising intelligent systems and sensors in the context of recent trends of emerging technologies.

## Figures and Tables

**Table 2:** Comparison of IoT-based technologies for smart cities.

Reference	Technology	Frequency	Data Rate	Range	Used For	Main Application Area
[22]	ZigBee	2.4 GHz	250 Kbps, 100 Kbps, 40 Kbps, 20 Kbps	10–100 m	Control and monitor applications	Home automation.
[23]	Actuator	100 Hz	10Mbps	—	Produces mechanical motion by converting the energy in a control signal	Smart Lighting, Air conditioning in home.
[24,25]	MQTT	10 Hz, 2 Hz, 1 Hz	1 Mbps	256 m	Interconnect devices	Transport System
[26,27]	Ad-hoc	2.4 GHz	10 Kbps	100 m	Helps in emergencies	Smart Health.
[28]	GPS	1575.42 MHz, 1227.6 MHz	50 Mbps	500–30 cm	Show precise location of an element	Waste Management System, Smart Home, Smart Parking.

**Table 3:** Strategies of cities around the world.

City	Strategies
Singapore	Establishing video consultations, monitor patients remotely, vehicle free city.
Oslo	Energy saving adjusting traffic lights, electric vehicles, free parking.
New York	WiFi-based charging stations, car sharing reduces emission and traffic congestion.
London	5G connectivity, fibre-optic coverage, lampposts, electric vehicle charging points.
Copenhagen	Smart parking, monitor air quality and traffic lighting, energy consumption.

**Table 5:** IoT challenges and solutions of smart city.

Challenge	Solution	Reference
Reliability	Decentralized and distributed architectures and decision making, Energy Efficient	[16]
Assuring data quality	Cost effective, Efficient Data gathered might be resolved	[24]
Security and Privacy	Prevent data leaks, Ensure that their sensitive data has private access, New authentication techniques, Encryption, Steer clear of theft and unethical manipulation	[93]
Smart Sensors	Enable measurement, inference, and comprehension of environmental indicators, Low power sensors, High efficiency, encouraging interoperability	[93,94]
Networking	Low power networks,	[95]
Big data	Scalable, Efficient, Big data processing centers that are centralized, give sensitive data data anonymity	[96,97]
Large Scale	Provide storage and computational capability to extract new information, Handle delay	[97]
